# Two-Dimensional Gel Electrophoresis-Based Proteomic Analysis Reveals N-terminal Truncation of the Hsc70 Protein in Cotton Fibers *In Vivo*

**DOI:** 10.1038/srep36961

**Published:** 2016-11-11

**Authors:** Chengcheng Tao, Xiang Jin, Liping Zhu, Hongbin Li

**Affiliations:** 1 College of Life Sciences, Key Laboratory of Agrobiotechnology, Shihezi University, Shihezi, 832003, China; 2 Institute of Tropical Biosciences and Biotechnology, Chinese Academy of Tropical Agricultural Sciences, Haikou, 571101, China

## Abstract

On two-dimensional electrophoresis gels, six protein spots from cotton ovules and fibers were identified as heat shock cognate 70 kD protein (Hsc70). Three spots corresponded to an experimental molecular weight (MW) of 70 kD (spots 1, 2 and 3), and the remaining three spots corresponded to an experimental MW slightly greater than 45 kD (spots 4, 5 and 6). Protein spots 1, 2 and 3 were abundant on gels of 0-day (the day of anthesis) wild-type (WT) ovules, 0-day fuzzless-lintless mutant ovules and 10-day WT ovules but absent from gels of 10-day WT fibers. Three individual transcripts encoding these six protein spots were obtained by using rapid amplification of cDNA ends (RACE). Edman degradation and western blotting confirmed that the three 45 kD Hsc70 protein spots had the same N-terminal, which started from the T271 amino acid in the intact Hsc70 protein. Furthermore, quadrupole time-of-flight mass spectrometry analysis identified a methylation modification on the arginine at position 475 for protein spots 4 and 5. Our data demonstrate that site-specific *in vivo* N-terminal truncation of the Hsc70 protein was particularly prevalent in cotton fibers, indicating that post-translational regulation might play an important role in cotton fiber development.

Cotton fiber is one of the longest single plant cells in the world, making it the ideal model of cell elongation^1^. Two-dimensional electrophoresis (2-DE) gel -based proteomic approaches have been widely used to investigate the protein-level molecular mechanism of cotton fiber development in the past two decades. The quantity of proteins such as ascorbate peroxidase (APX), UDP-4-keto-6-deoxy-D-glucose 3, 5-epimerase 4-reductase (UER), and sucrose synthase (Susy) as well as ROS homeostasis^2^,^3^,^4^,^5^,^6^ are important during cotton fiber development. These findings supplement transcriptional-level research on cotton fiber development, especially on post-translational modification mechanisms^7^,^8^. Gel-free high-throughput mass spectrometry (MS) approaches have been applied in recent years to identify proteins on a larger scale with higher sensitivity and reveal new aspects of the protein-level regulatory mechanism of cotton fiber development^9^,^10^,^11^,^12^,^13^. However, 2-DE technology is irreplaceable because it yields visualization maps of protein profiles, which provide information on the abundance of proteins and reliable evidence for existing protein isoforms^14^.

Hsc70 proteins are ATP-dependent molecular chaperones that bind unfolded proteins via various biological processes such as *de novo* protein folding, protein refolding and protein translocation^15^,^16^,^17^,^18^. Hsc70s have three domains: an N-terminal nucleotide binding domain (NBD), a substrate binding domain (SBD) and a C-terminal helical domain (Lid domain). The NBD and SBD domains are highly conserved, while the helical lid domain diverges strongly between eukaryotic and prokaryotic species^19^. The mechanism of Hsp70 has been well described^20^. Hsp70 has a low affinity for unfolded proteins in the ATP-bound state. After co-chaperones assist the ATP hydrolysis process, the substrate is efficiently bound by ADP-Hsp70. All three domains are likely involved in this process^21^,^22^,^23^. The co-chaperones of Hsc70 include DnaJ and nucleotide exchange factors (NEFs), which trigger the dissociation of ADP from Hsc70, resetting the ATP hydrolysis cycle^20^. New types of Hsc70 co-chaperones or inhibitors have been reported^24^,^25^,^26^, which indicates that in-depth investigations on the mechanism of Hsc70 are necessary.

*In vivo*, many proteins need to be processed from their precursors to become mature proteins. Protein truncation, particularly N-terminal signal peptide truncation^27^, is one of the most common post-translational processes of proteins. Many proteins, such as 5-aminolevulinate synthase (ALAS), activating transcription factor 6 (ATF6) and eukaryotic translation elongation factor 1Bα (eEF1Bα) are functional or activated after N-terminal truncation^28^,^29^,^30^. The N- and C-terminal truncation of proteins improves the thermostability of some proteins, such as endo-β-1, 4-glucanase and 1, 4-α-D-glucan glucanohydrolase (α-Amylase, Amy703)^31^,^32^. The truncation of Hsp/Hsc family proteins has been used to determine the functions of different components of Hsp/Hsc proteins; however, the *in vivo* native truncation of Hsc70s has not yet been reported^19^,^33^.

Here, we report the *in vivo N*-terminal truncation of Hsc70 in cotton fibers, which was originally discovered in 2-DE gels. Matrix-assisted laser desorption ionization time of flight (MALDI-TOF) MS, Edman degradation and western blotting confirmed the amino acid site of truncation. Quadrupole time of flight mass spectrometry (Q-TOF MS) identified the methylation of R475 in two of the three truncated protein spots.

## Material and Methods

### Cotton plants

Upland cotton *Gossypium hirsutum* (Gh) acc. Xuzhou142 wild type (WT) and the corresponding fuzzless-lintless mutant (*fl*) were grown in a soil mixture in a fully automated greenhouse with 60% relative humidity at 34 °C in the light and 28 °C in the dark (12 h light/dark cycle). Cotton bolls of the 0-day WT (on the day of anthesis, WT-0), 0-day *fl* (*fl*-0), 10-day WT (WT-10), 10-day *fl* (*fl*-10) were detached from the 3^rd^ and 5^th^ fruit spurs from 10 am to 12 am, to avoid unexpected changes in the protein expression level involving development and the circadian clock. To separate the 10-day fibers (WT-10-F), ovules (WT-10-O) were fixed with tweezers and fibers were pulled off with another set of tweezers. All plant materials were frozen and stored in liquid nitrogen immediately after harvesting.

### Protein extraction and 2-DE

Total protein extraction was performed as described previously^2^. The concentrations of protein samples were determined by a Bradford assay. Approximately 1.2 mg of proteins from each sample were independently loaded onto 24 cm immobilized pH gradient (IPG) strips (GE Healthcare Life Sciences, Pittsburgh, PA, USA) with linear pH gradients from 4–7. Isoelectric focusing and sodium dodecyl sulfate polyacrylamide gel electrophoresis (SDS-PAGE) were performed as previously described^34^. The gels were visualized by GAP staining methods^35^ and analyzed using Image Master 2D Platinum Software (Version 5.0, GE Healthcare Life Sciences). Three biological duplicates were performed for each cotton material.

### Identification of protein spots by MS/MS

Proteins spots were excised and digested with modified bovine trypsin (cat. no. 11418025001, Roche, Basel, Switzerland) as previously reported^3^. Mass spectra of trypsin-digested peptide extracts were recorded on an AB SCIEX MALDI-TOF/TOF 5800 system (AB SCIEX, Framingham, MA, USA) with a laser wavelength of 349 nm. Unsing an in-house MASCOT server (Matrix Science, Boston, MA, USA), we searched for all spectra in a self-constructed database derived from the original Gh genome and expressed sequence tags^36^,^37^,^38^ that included 77,051 protein sequences. All six protein spots were considered to be successfully identified onlyif peptide counts with 95% confidence >5 and peptide coverage >20%.

### 5′-RACE and western blotting

Total RNA was extracted as previously described^39^. We conducted 5′-RACE with a GeneRacer kit (Invitrogen, Waltham, MA, USA), following the manufacturer’s instructions. The PCR product of nest-PCR was sequenced and aligned to the known portions of Hsc70 genes. Three independent sequences were obtained via 5′-RACE, validated by sequencing, and submitted in full to GenBank (detailed information available under acc. nos. FJ415196.1, FJ415194.1 and XM_016868691.1).

Twenty micrograms of proteins of WT-0, *fl*-0, WT-10, WT-10-O, WT-10-F, *fl*-10 and prokaryotic expressed GhHsc70 were loaded per lane on SDS-PAGE gels for blotting. Commercially available antibodies against GAPDH (cat. no. ab9485, Abcam, Santa Cruz, CA, USA) and the conserved SBD domain of Hsc70 (ab137808, Abcam) were used. Western blotting experiments were then performed as reported previously^40^.

### *In vitro* expression of Hsc70

The 2308 bp coding region of *GhHsc70–1* was amplified using primers with additional restriction sites for *Bam*HI and *Kpn*I at the 5′ end. The PCR fragment was cloned into the pBlueScript SK vector using *Bam*HI and *Kpn*I. The resulting construct was then transformed into *E. coli* BL21 (DE3). The transformed bacteria were cultured in liquid Luria-Bertani (LB) medium containing 50 μg/ml kanamycin with stable shaking at 37 °C. Isopropyl-1-thio-β-D-galactoside (IPTG) was added to the medium to a final concentration of 0.4 mM, with a cell density between 0.6 and 0.8 and an optical density of 600 nm. The cultures were harvested by centrifuging at 5000 g at 4 °C for 20 min after additional incubation at 37 °C for 4 h. The pellets were suspended in a binding buffer (50 Mm Tris-HCL, 0.5 M NaCl, 1% Triton X-100, pH 8.0). The lysate was centrifuged at 10,000 g for 10 min at 4 °C after sonication. The fractions containing the recombinant GhHSC70–3 were eluted from the column after the supernatant was loaded on the Ni-charged His-Bind column. The objective peak fractions were determined by SDS-PAGE and used for western blotting analysis as a positive control.

### Q-TOF MS/MS

ESI-MS/MS was performed for the purified tryptic digests of spots 4, 5 and 6 using a quadrupole time-of-flight mass spectrometer (Micromass, Manchester, UK) equipped with a Z-spray source. The peptides were loaded by nanoelectrospray with gold-coated borosilicate glass capillaries (Micromass), with the spray voltage set to 800 V. The collision energy varied from 14 to 40 V according to the mass and charge state of the peptides. Tandem MS peak lists were uploaded to the Mascot MS/MS Ions Search program on the Matrix Science public website. A peptide tolerance of ±2.0 Da for the precursor ions and an MS/MS tolerance of ±1.2 Da for the fragment ions were set.

## Results

### Six Hsc70 protein spots were identified on 2-DE gels

**Figure 1:**
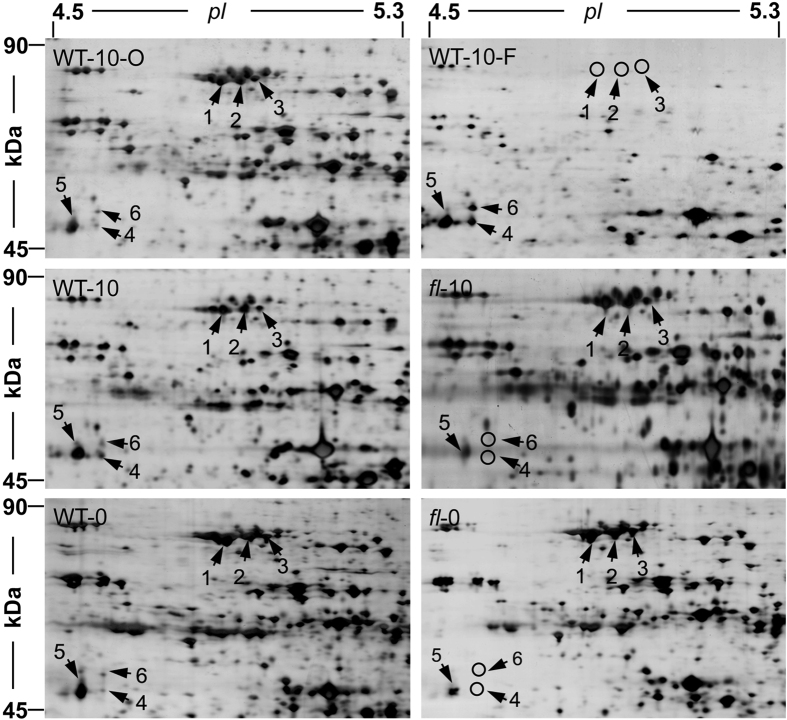
Enlarged 2-DE gel regions of six Hsc70 protein species. Enlarged 2-DE map regions of six different cotton samples are shown: 10-day WT ovules (WT-10-O), 10-day WT fibers (WT-10-F), 10-day WT ovules and fibers (WT-10), 10-day *fl* ovules (*fl*-10), 0-day WT ovules (WT-0) and 0-day *fl* ovules (*fl*-0). The shown regions had a *pI* range from 4.5 to 5.3 (indicated on the top) and a MW range from 45 kDa to 90 kDa (indicated on the left). Six Hsc70 protein spots are marked with arrows in each 2-DE map. Circles indicate vanished protein spots. Representative 2-DE gels are shown in Supplementary Fig. 1, in which the enlarged regions in Fig. 1 are framed.

On 2-DE gels of different cotton tissues, we observed six protein spots identified as Hsc70 by MALDI-TOF (with matched peptides listed in Table 1). Three of these protein spots had an experimental MW of 70 kD (spots 1, 2 and 3) and the other three had an experimental MW of slightly larger than 45 kD, which was significantly different from the calculated MW (spots 4, 5 and 6). Interestingly, spots 1, 2 and 3 had opposite protein abundance levels when compared to spots 4, 5 and 6 (Fig. 1). The 2-DE gel regions shown in Fig. 1 were framed in the corresponding 2-DE gels (Supplementary Fig. S1). Protein spots 1, 2 and 3 accumulated in WT-0, *fl*-0, WT-10, *fl*-10 and WT-10-O, but were absent in WT-10-F. Protein spot 5 was present in WT-0, *fl*-0, WT-10, *fl*-10 and WT-10-O with different expression levels, while it was significantly accumulated in WT-10-F. Notably, protein spots 4 and 6 were totally absent in both *fl*-0 and *fl*-10 but were present in WT-0, WT-10 and WT-10-O with lower expression levels. A much higher expression level was found in WT-10-F.

**Figure 2:**
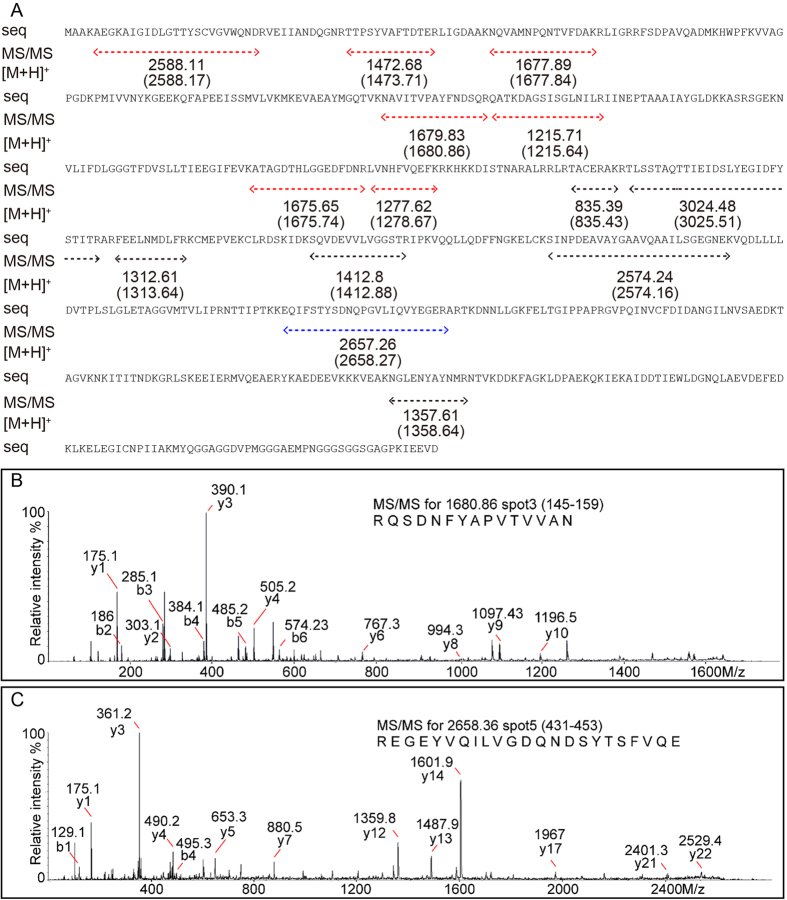
Representative MS/MS identification information of Hsc70 protein spots 3 and 5. (**A**) Sequence of the intact Hsc70 protein (coded by transcript *GhHsc-3*). Peptides identified by MS/MS are labeled with dashed lines: red lines, peptides only identified in spot 3; blue lines, peptides only identified in spot 5; black lines, peptides identified in both spots 3 and 5. The m/z values ([M+H]^+^) are also indicated under the dashed lines. Numbers represent the calculated m/z for the corresponding peptides, while bracketed numbers represent the observed m/z in our MS/MS experiment. (**B**,**C**). Representative peak-view of MS/MS data for the 1680.86 precursor of spot 3 and 2658.36 precursor of spot 5, respectively.

Due to their abundance, these protein spots were selected as corresponding to differentially abundant proteins (DAP) and were identified by MALDI-TOF after in-gel digestion by trypsin. All six protein spots were identified as Hsc70 using the protein search engine MSCOT version 2.5.1. The MS-identified peptides of protein spots 3 and 5 were shown by colored lines (red for spot 3, blue for spot 5 and black for both), with the experimental (top) and calculated (bottom, blanked) m/z values (Fig. 2A). Notably, peptides that only matched protein spot 3 (red lines) were scattered only in the region of amino acids (aa) 1–252, while peptides matched by both spots 3 and 5 (black lines) and the specific matched spot 5 (blue line) were distributed in the region of 271–652 aa, which indicated that the 45 kD protein spot 5 might be a truncated fragment of Hsc70. Representative peak views of the MS/MS map of protein spot 3 (Fig. 2B) and protein spot 5 (Fig. 2C) are also shown. Furthermore, the matched peptides of protein spots 1 and 4 are also shown in online Supplementary Fig. S2A, and the representative MS/MS peak views of spots 1 and 4 are shown in Supplementary Fig. S2B,C, respectively. The same information for protein spots 2 and 6 is shown in Supplementary Fig. S3.

### Sequence and expression analyses of GhHsc70s

In accordance with information for identified peptides, RACE technology was used to obtain transcripts coding for the 6 mentioned protein spots. Three individual transcripts were cloned: *GhHsc70-1* (2308 bp, GenBank acc. No. FJ415196.1), *GhHsc70-2* (2294 bp, GenBank acc. No. FJ415194.1) and *GhHsc70-3* (2190 bp, GenBank acc. No. XM_016868691.1), encoding 648 aa, 647 aa and 652 aa Hsc70 family proteins, respectively. The corresponding relationships between the three transcripts and six protein spots were determined by MS matched peptides and sequence analysis, with *GhHsc70-3* encoding protein spots 3 and 5, *GhHsc70-1* encoding protein spots 1 and 4, and *GhHsc70-2* encoding protein spots 2 and 6, respectively (Fig. 2, Supplementary Figs S2 and S3).

**Figure 3:**
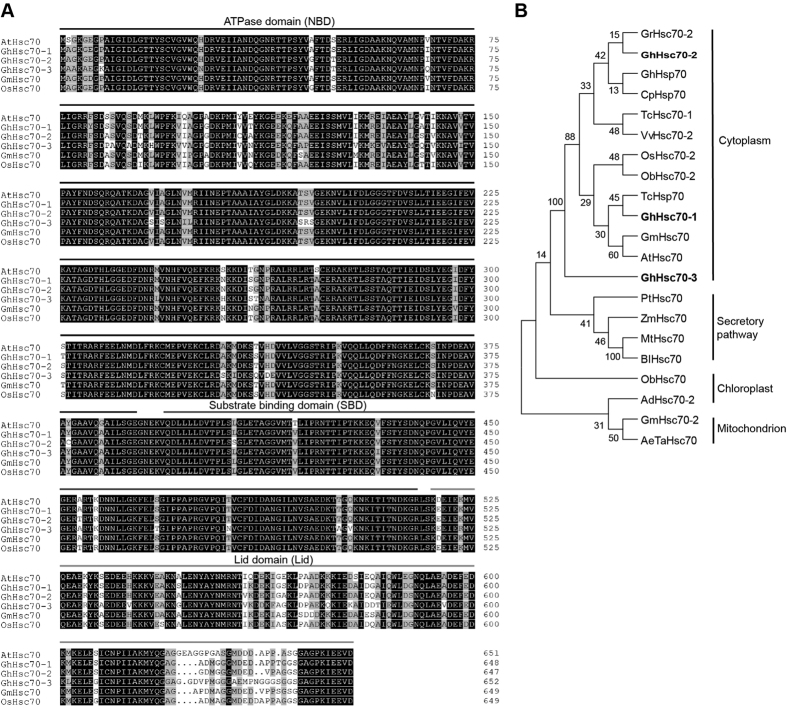
Sequence analysis of Hsc70 transcripts. (**A**) Multiple sequence alignment of representative Hsc70 proteins from *Arabidopsis thaliana* (At), *Gossypium hirsutum* (Gh), *Glycine max* (Gm) and *Oryza sativa* (Os). The conserved domains of the Hsc protein family are indicated: with solid lines. (**B**) Phylogenetic tree of the Hsc70 protein family. Twenty-one members of the Hsc70 family collected from 15 plants were used to construct an NJ phylogenetic tree using Molecular Evolutionary Genetics Analysis (MEGA) software. The bootstrap was set to 1000 replicates. The Hsc70s were divided into four sub-families: cytoplasm, secondary pathway, chloroplast and mitochondrion, which indicated different sub-cellular locations of these Hsc70s. Three *GhHsc70* genes cloned from *Gossypium hirsutum* are labeled in bold. Numbers beside the branches indicate the bootstrap values that supported the adjacent nodes. Ad, *Arundo donax*; AeTa, *Aegilops tauschii*; At, *Arabidopsis thaliana*; Bl, *Betula luminifera*; Cp, *Chimonanthus praecox*; Gh, *Gossypium hirsutum*; Gr, *Gossypium ramondii*; Gm, *Glycine max*; Mt, *Medicago truncatula*; Ob, *Oryza brachyantha*; Os, *Oryza sativa*; Pt, *Populus trichocarpa*; Tc, *Theobroma cacao*; Vv, *Vitis vinifera*; Zm, *Zostera marina*.

Multiple sequence alignment demonstrated that all protein sequences deduced from the three transcripts included known conserved domains of the Hsc70 protein family (Fig. 3A). The identity of the Hsc70 family was 94.82%, confirming that the three individual transcripts encoded three Hsc70 protein family members in *Gossypium hirsutum*. A phylogenetic tree indicated that all three GhHsc70s belonged to the sub-family of cytoplasmic Hsc70s (Fig. 3B). They were distributed into three different branches: GhHsc70-1 had the greatest similarity to *Arabidopsis thaliana* Hsc70 (AtHsc70); GhHsc70-2 was clustered with *Gossypium ramondii* Hsc70-2; and GhHsc70-3 branched earliest in the cytoplasmic sub-family (Fig. 3B).

**Figure 4:**
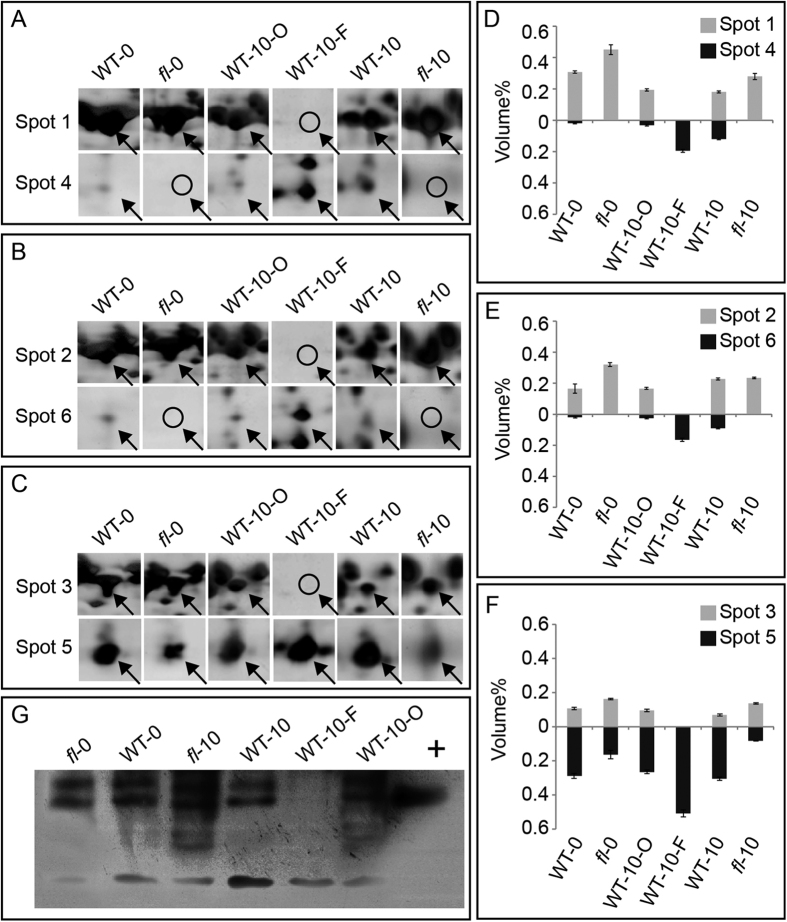
Quantitative analysis of Hsc70 proteins in cotton ovules and fibers. (**A**) Enlarged protein spot 1 and spot 4 of 2-DE maps of WT-0, *fl*-0, WT-10-O, WT-10-F, WT-10 and *fl*-10. Spots are marked with arrows in each enlarged 2-DE map. Circles indicate the vanished protein spots. (**B**,**C**). Similar information for spots 2 and 6 and spots 3 and 5, respectively. **(D**–**F**). Percentages of gray volume (Volume%) for protein spots 1 and 4, protein spots 2 and 6, and protein spots 3 and 5, respectively. (**G**) Western blot of Hsc proteins in cotton samples of *fl*-0, WT-0, *fl*-10, WT-10, WT-10-F and WT-10-O. The symbol + indicates the positive control, which was prokaryote expressed GhHsc70.

To further examine the expression patterns of the three transcripts, digital signal analysis of 2-DE protein spots and western blotting assays were performed. The 2-DE gel regions of the six protein spots were enlarged to highlight the negative co-relationship between the three pairs of spots (Fig. 4A–C). The percent gray volume of all six protein spots was also determined and indicated the negative correlativity between spot 1 and spot 4 (Fig. 4D), spot 2 and spot 6 (Fig. 4E), and spot 3 and spot 5 (Fig. 4F).

Moreover, both the 70 kD and 45 kD bands were detected by western blotting in WT-0, *fl*-0, WT-10, WT-10-O and *fl*-10, whereas only a 45 kD band could be detected in WT-10-F (Fig. 4G, Supplementary Fig. S4). Only one 70 kD band was detected in the positive Hsc70 lane (Fig. 4G), which was loaded with 5 μg of prokaryote expressed Hsc70; this result indicated extremely high antibody specificity. The results of western blotting confirmed the changing pattern of the abundance of protein spots observed on the 2-DE gels. In addition, western blotting using 2-DE gels of WT-10-F produced a positive result, further confirming that protein spots 4, 5 and 6 are truncated fragments of GhHsc70s (Supplementary Fig. S5).

### Edman degradation and Q-TOF MS of the three 45 kD protein spots

**Figure 5:**
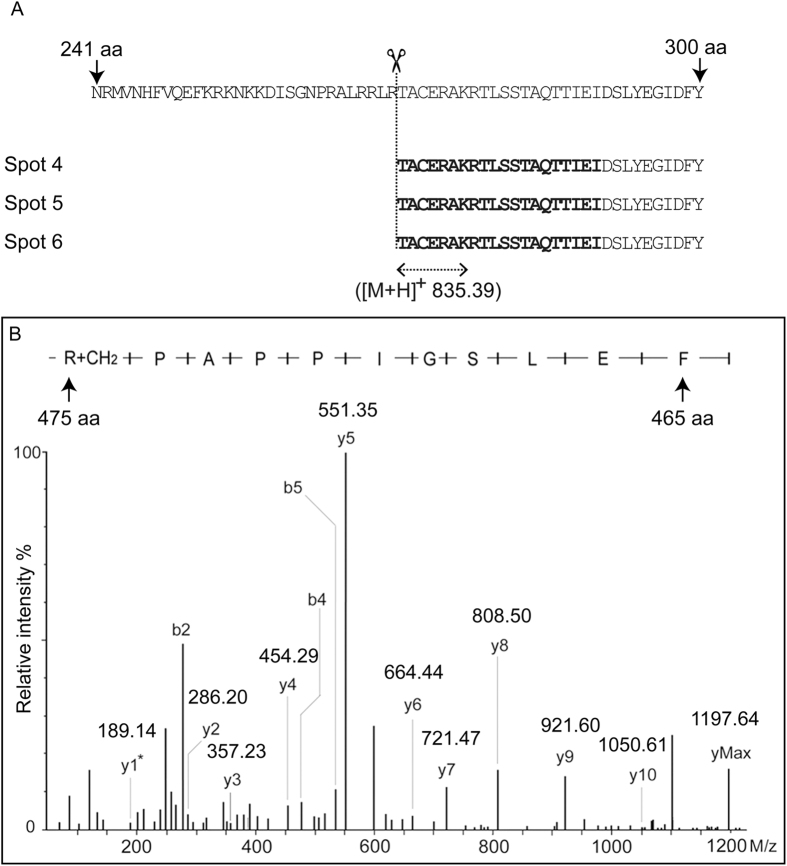
Edman degradation and Q-TOF analyses of spots 4, 5 and 6. (**A**) Edman degradation of protein spots 4, 5 and 6 showed that these proteins share the same 20 N-terminal amino acids, which indicated that Hsc70 may be truncated at 271 amino acids. The twenty sequenced amino acids are bolded. The peptide TACERAK detected by MS/MS and its m/z values (835.39) are indicated. (**B**) Representative CID MS/MS peak view of the precursor 599.35. The sequence identified was the 465–475 peptide of GhHsc70, with the b and y ions shown. The Y1 ion had a MW of 189.14 kDa, which was 13.98 kDa greater than the theoretical MW of arginine, indicating a potential methylation modification on R475.

To validate the cleavage site of the 45 kD Hsc70 protein spots, the sequence of the 20 N-terminal amino acids was determined by Edman degradation. All three truncated protein spots had exactly the same N-terminal amino acid sequence: TACERAKRTLSSTAQTTIEI (Fig. 5A). The ±30 aa sequence (241–300 aa) of the cleavage site T275 residue was on the top, while the TACERAK peptide, which had a calculated m/z of 835.39, was detected by MS in all three 45 kD protein spots (Fig. 2A, Supplementary Figs S2A and S3A). This result supported the results of Edman degradation.

Q-TOF was performed to determine the possible modification of protein spots 4, 5 and 6 to explain the different experimental isoelectric point (*pI*) and MW values between protein spots 4, 5 and 6. The representative CID MS/MS peak view of the precursor 599.35 is shown in Fig. 5B. The sequence identified is the 465–475 peptide of GhHsc70, with the b and y ions shown. The C-terminal amino acid corresponding to the Y1 ion had a MW of 189.14, which is 13.98 larger than the theoretical MW of arginine; this value indicated the methylation modification on R475. Interestingly, a methylation modification on R475 was detected in spot 4 and spot 5, but not in spot 6, which indicated that the post-translational modification may cause the 45 kD fragment of GhHsc70 to split into three individual protein spots on 2-DE gels.

## Discussion

Since it was invented in the 1980 s, 2-DE gel technology has been the predominant proteomic approach. However, it is limited due to its low sensitivity (usually no more than 2,000 protein spots can be detected) and its low identification rate of less abundant protein spots. In recent years, gel-free high-throughput MS technologies have been widely used instead of 2-DE gels, benefitting from their high sensitivity and high throughput for identifying proteins^7^,^8^,^9^,^10^,^11^,^12^,^13^. However, 2-DE gel technology can still provide advantages that are not available for gel-free approaches, such as visualization maps of protein profiles, information about the MW and *pI* of individual protein spots and, most importantly, reliable evidence for existing protein isoforms^14^. This work describes the application of 2-DE gel technology to investigate the post-translational regulation of functional proteins.

Many proteomic studies have identified several protein spots on 2-DE maps as exactly the same protein, called protein species^41^,^42^. Researchers are aware that these protein spots may represent different modifications of the same proteins (especially for protein spots with experimental MWs and *pI values* that differ from the calculated MWs and p*I*). However, few works have discussed this phenomenon in depth. On 2-DE gels, we noticed dramatic changes in the 45 kD protein spots, which were identified as Hsc70 (Figs 1 and 4). We designed experiments to validate this identification and illustrate the post-translational truncation and modification of Hsc70 proteins in cotton fibers, which may play roles in cotton fiber development. This work provides a feasible procedure for 2-DE gel-based proteomic investigations, benefiting from the irreplaceable characteristics of 2-DE technology compared to gel-free MS technologies.

The six Hsc70 protein spots reported herein had very different MWs (70 kD for spots 1, 2 and 3; slightly greater than 45 kD for spots 4, 5 and spots 6). Interestingly, the 70 kD and 45 kD protein spots had opposite accumulation patterns, which indicated that they may be interconverted into each other in different cotton tissues (Fig. 4A–C and E–G). RACE and sequence analyses showed that the six protein spots of Hsc70 were coded by three individual transcripts: *GhHsc70-1*, *GhHsc70-2* and *GhHsc70-3* (Fig. 3). BLAST against the genome sequence of *Gossypium hirsutum* (tetraploid cotton, AADD genome)^36^,^37^ showed that these three transcripts had two copies each on the A sub-genome and D sub-genome (*GhHsc70-1* mapped to Gh_A06G1477 and Gh_D06G1814; *GhHsc70-2* mapped to Gh_A11G2910 and Gh_D11G3296; *GhHsc70-3* mapped to Gh_A13G2046 and Gh_D13G2447; Supplementary Fig. S6).

The Edman degradation confirmed that the N-termini of the three 45 kD protein spots were exactly the same, which indicated that they were site-specifically truncated by unknown factors (Fig. 5A). The cleavage site was located between R274 and T275, which is possibly cleaved by several endogenous proteases in plants^43^. Notably, protein spot 6 had a MW slightly greater than that of spots 4 and 5 (Fig. 1). Q-TOF analysis detected methylation modification on R475 of spots 4 and 5, but not for spot 6 (Fig. 5B), which provided a possible explanation for the difference in MW between the three truncated GhHsc70 protein spots. We observed that protein spots 4 and 6 were totally absent from 2-DE gels of *fl*-0 and *fl*-10 but were present at lower abundances in gels of WT-0, WT-10 and WT-10-O; however, these protein spots exhibited the greatest abundance on 2-DE gels of WT-10-F. This finding might indicate that enzymes involved in the post-translational modification of Hsc70 isoforms corresponding to spots 4 and 6 had complete loss of function in *fl* and partial loss of function in WT ovules. Hypothetically, the Hsc70 isoforms corresponding to spots 4 and 6 could be important for cotton fiber development. Further studies may provide more solid evidence to clarify this phenomenon.

Hsc70 was reported as a 70 kD constitutive heat shock cognate protein chaperone. Artificial truncation of the N-terminal ATPase domain of CeHsc70 resulted in a partial reduction of the ATP turnover rate, but it was not sufficient to block it. However, the helix Lid domain has been reported to influence the ATP turnover rate and cofactor affinities. Truncation of the C-terminal Lid domain will alter the rate-limiting step of the hydrolysis cycle^19^. Further evidence is still needed to determine whether the 45 kD form of GhHsc70 identified herein is functional or simply the intermediate product of the degradation of the Hsc70 protein. The 45 kD form of Hsc70 lost its 274 N-terminal amino acids, which is the ATPase domain of intact Hsc70. Therefore, if the 45 kD form of Hsc70 does function, it is only involved in substrate binding and hydrolysis.

In conclusion, our study provides a feasible procedure to utilize the advantages of 2-DE gel technology compared to gel-free high-throughput approaches. The investigation of the six GhHsc70 protein spots showed that these protein spots were coded by three individual transcripts and were site-specifically truncated at T275, particularly in cotton fiber tissue. Q-TOF analysis detected a methylation modification on R475 of protein spot 4 and spot 5. This study reports the site-specific truncation of GhHsc70 and the methylation of truncated Hsc70 protein in repidly elongating cotton fibers *in vivo* and provides possible explanations for this phenomenon.

## Additional Information

**How to cite this article**: Chengcheng, T. *et al*. Two-Dimensional Gel Electrophoresis-Based Proteomic Analysis Reveals N-terminal Truncation of the Hsc70 Protein in Cotton Fibers *In Vivo*. *Sci. Rep*. **6**, 36961; doi: 10.1038/srep36961 (2016).

**Publisher’s note:** Springer Nature remains neutral with regard to jurisdictional claims in published maps and institutional affiliations.

## Supplementary Material

Supplementary Information

## Figures and Tables

**Table 1:** Detailed information of the mapped peptides for six Hsc70 protein spots.

Observed m/z	Calculated m/z	Position	Peptide sequence	Spot No.
2844.42	2844.39	2–28	AGKGEGPAIGIDLGTTYSCVGVWQHDR	1, 2
2588.17	2588.11	5–28	GEGPAIGIDLGTTYSCVGVWQHDR	2, 3
1473.70	1473.62	39–51	TTPSYVAFTDSER*	1, 2, 3
1677.84	1677.89	60–74	NQVAMNPINTVFDAK	2, 3
1680.83	1680.75	145–159	NAVVTVPAYFNDSQR*	1, 2, 3
1643.85	1643.81	160–175	QATKDAGVIAGLNVMR	2, 3
1215.64	1215.71	164–175	DAGVIAGLNVMR*	1, 2, 3
1787.99	1787.86	176–192	IINEPTAAAIAYGLDKK	1, 2,
1675.74	1675.65	227–242	ATAGDTHLGGEDFDNR	1, 2, 3
1278.70	1278.61	243–252	MVNHFVQEFK*	1, 2, 3
1450.72	1450.66	243–253	MVNHFVQEFKR	1, 3
835.39	835.43	271–277	TACERAK	1, 2, 3, 4, 5, 6
1540.83	1540.75	306–317	ARFEELNMDLFR*	1, 2, 3, 5, 6
1329.66	1329.61	308–317	FEELNMDLFR	2, 3, 4, 5
1441.78	1441.71	308–318	FEELNMDLFRK	2, 3, 5, 6
1786.97	1786.89	332–348	MDKSSVHDVVLVGGSTR	1, 2, 3, 4, 6
1412.80	1412.88	335–348	SSVHDVVLVGGSTR*	1, 2, 3, 4, 5, 6
1967.09	1967.01	352–367	VQQLLQDFFNGKELCK	1, 3, 5, 6
2574.24	2574.16	368–393	SINPDEAVAYGAAVQAAILSGEGNEK	1, 2, 3, 4, 5, 6
2658.33	2658.25	431–453	EQVFSTYSDNQPGVLIQVYEGER*	2, 4, 5, 6

^*^These peptides were confirmed by MS/MS.
