# Supplementary material for: Two-Dimensional Gel Electrophoresis-Based Proteomic Analysis Reveals N-terminal Truncation of the Hsc70 Protein in Cotton Fibers In Vivo
Source: Sci Rep. 2016 Nov 11;6:36961. doi: 10.1038/srep36961 (PMC5105075; doi:10.1038/srep36961)

Supplementary Information of SREP-16-27051

Title: Two-Dimensional Gel Electrophoresis-Based Proteomic Analysis Reveals N-terminal Truncation of the Hsc70 Protein in Cotton Fibers *In Vivo*

Authors: Tao Chengcheng[1,2*], Jin Xiang[1,2*], Zhu Liping[1], Li Hongbin[1#]

1. College of Life Sciences, Key Laboratory of Agrobiotechnology, Shihezi University, Shihezi, 832003, China
2. Institute of Tropical Biosciences and Biotechnology, Chinese Academy of Tropical Agricultural Sciences, Haikou, 571101, China

Supplementary Table S1 Primers used in this study.

Primer name	Primer sequence
GhHsc70-1 sense	5'-CGGGATCCTCTTAACAAGCAACACTCGTCTC-3'
GhHsc70-1 anti-sense	5'-GGGGTACCTATAATTCTTAAAACGGGGAACA-3'
RACE universal primer long	5’-CTAATACGACTCACTATAGGGCAAGCAGTGGTATCAACGCAGAGT-3’
RACE universal primer short	5’-CTAATACGACTCACTATAGGGC-3’
RACE NEST primer	5’-AAGCAGTGGTATCAACGCAGAGT-3’
GhHsc70-1 5’RACE	5'-CCATAGATGAGATTTCCTCGGCAGC-3'
GhHsc70-2 5’RACE	5'-CTGTAGACCCAAGGTAAGCCTCAGC-3'
GhHsc70-3 5’RACE	5'-CTCGGCAACCTCCTTCATCTTGAC-3'

Supplementary Figure Legends

Figure S1. Representative silver stained 2-DE maps for WT-0, *fl*-0, WT-10-O, WT-10-F, WT-10, and *fl*-10. The MW (kDa) and *pI* scales are indicated. The regions shown in Figure 1 are framed.

Figure S2. Representative MS/MS identification of Hsc70 protein spots 1 and 4. (**A**) Sequence of the intact Hsc70 protein (coded by transcript *GhHSC-1*). Peptides identified by MS/MS are labeled with dashed lines: red lines, peptides only identified in spot 1; blue lines, peptides only identified in spot 4; black lines, peptides identified in both spots 1 and 4. The m/z value ($[M+H]^+$) is also indicated under the broken lines. Numbers represent the calculated m/z values for the corresponding peptides, while bracketed numbers represent the observed m/z values in our MS/MS experiment. (**B**) and (**C**), representative peak-view of MS/MS data for the 1473.71 precursor of spot 1 and 3025.58 precursor of spot 4, respectively.

Figure S3. Representative MS/MS identification information of Hsc70 protein spots 2 and 6. (**A**) Sequence of the intact Hsc70 protein (coded by transcript *GhHSC-2*). Peptides identified by MS/MS are labeled with dashed lines: red lines, peptides only identified in spot 2; blue lines, peptides only identified in spot 6; black lines, peptides identified in both spots 2 and 6. The m/z value ($[M+H]^+$) is also indicated under the dashed lines. Numbers represent the calculated m/z values for the corresponding peptides, while bracketed numbers represent the observed m/z values in our MS/MS experiment. (**B**) and (**C**), representative peak views of MS/MS data for the 1278.67 precursor of spot 2 and 1540.78 precursor of spot 6, respectively.

Figure S4. Full length western blotting gel of GhHsc70. The representative full length western blotting gel of GhHsc70 are shown.

Figure S5. Western blotting of GhHsc70 on a 2-DE gel of WT-10-F. The representative western blotting of GhHsc70 on a 2-DE gel of WT-10-F. Spots 4, 5 and 6 are labeled with arrows. The *pI* and MW ranges are indicated.

Figure S6. Multiple sequence alignment of RACE cloned Hsc70 transcripts and the corresponding genome sequences. (**A**) multiple sequence alignment of *Hsc70-1* and Gh_A06G1477 and Gh_D06G1814. (**B**) multiple sequence alignment of *Hsc70-2* and Gh_A11G2910 and Gh_D11G3296. (**C**) multiple sequence alignment of *Hsc70-3* and Gh_A13G2046 and Gh_D13G2447.

Supplementary Figure S1

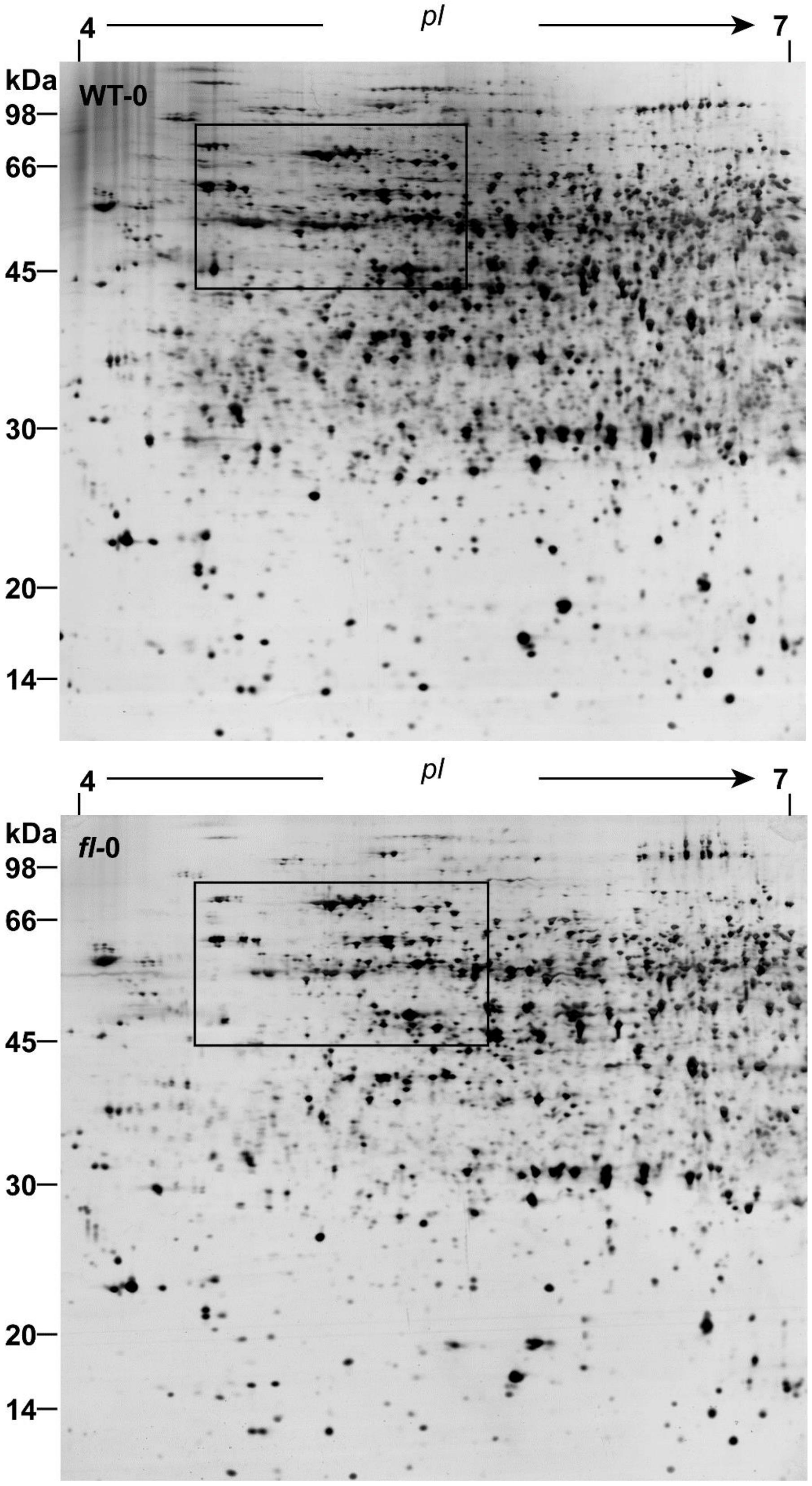

Supplementary Figure S1 (continued…)

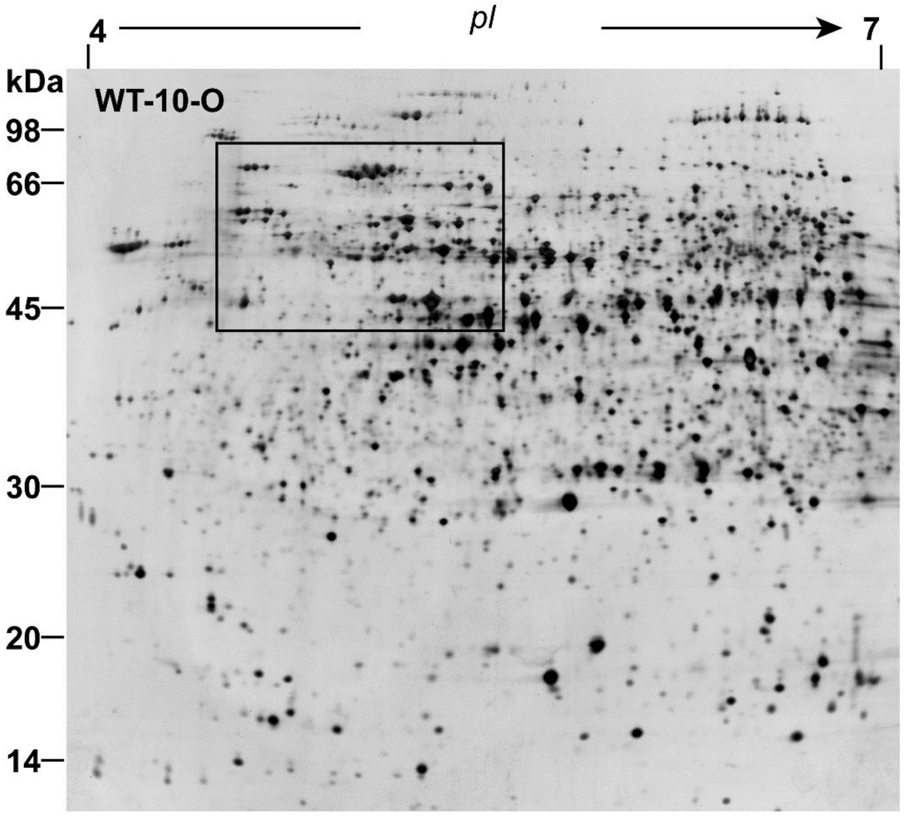

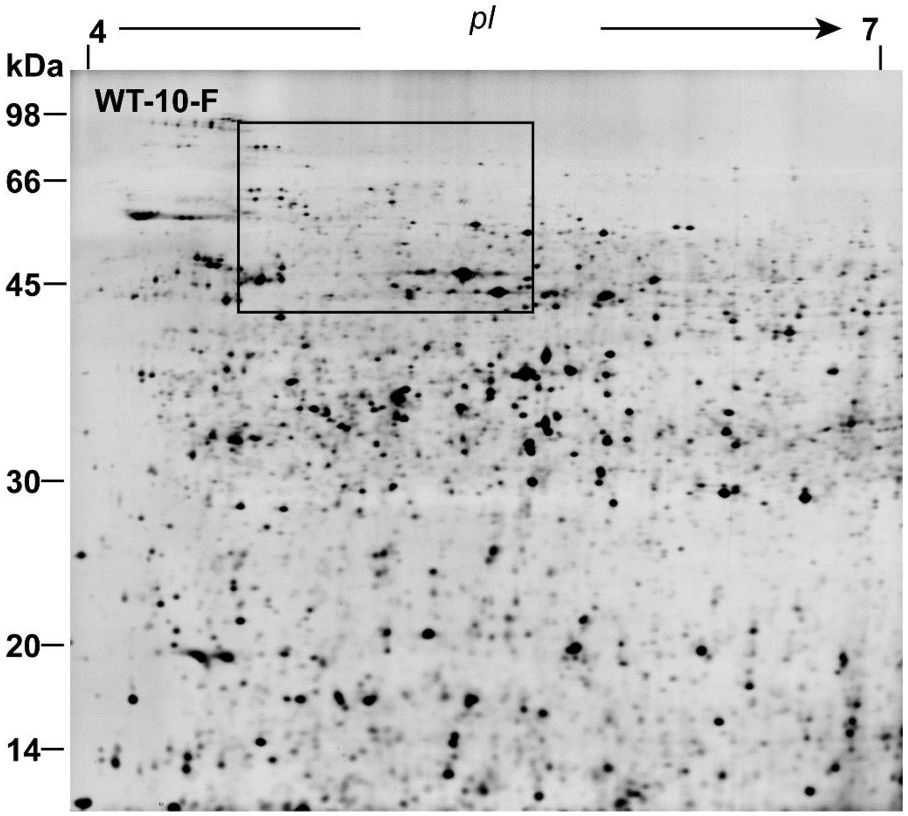

Supplementary Figure S1 (continued…)

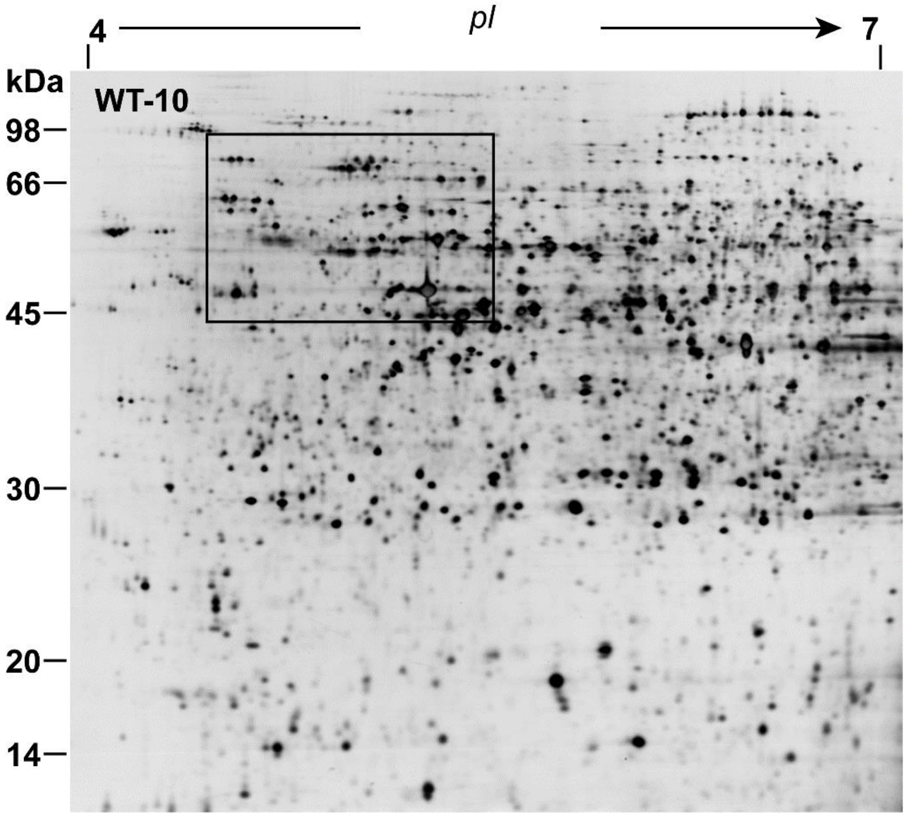

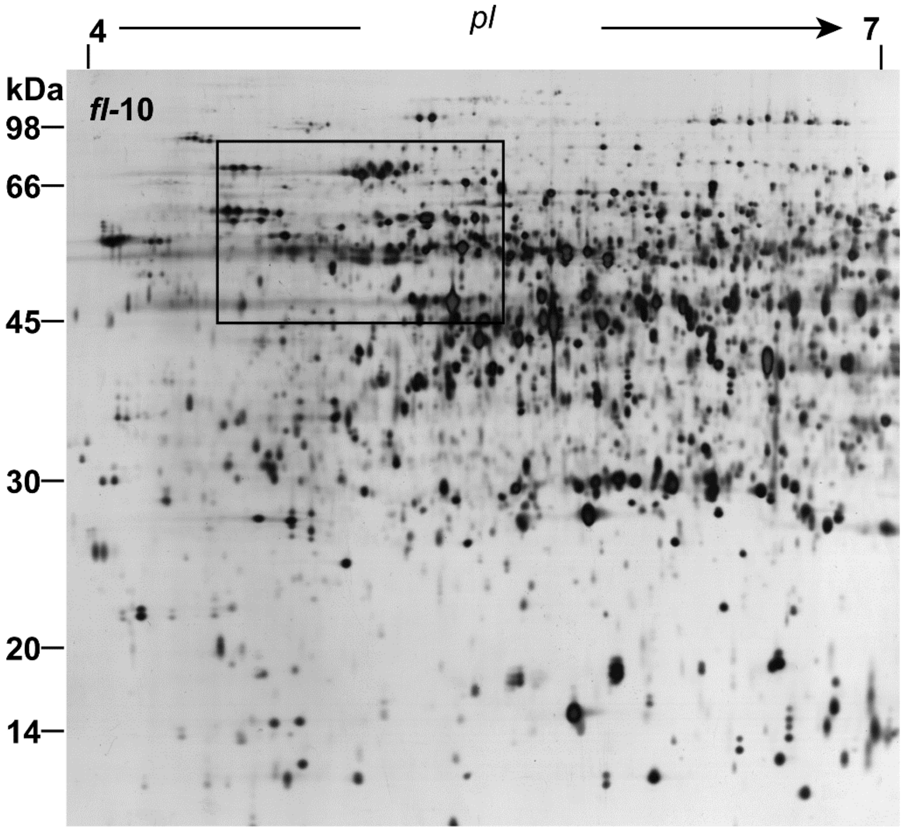

Supplementary Figure S2

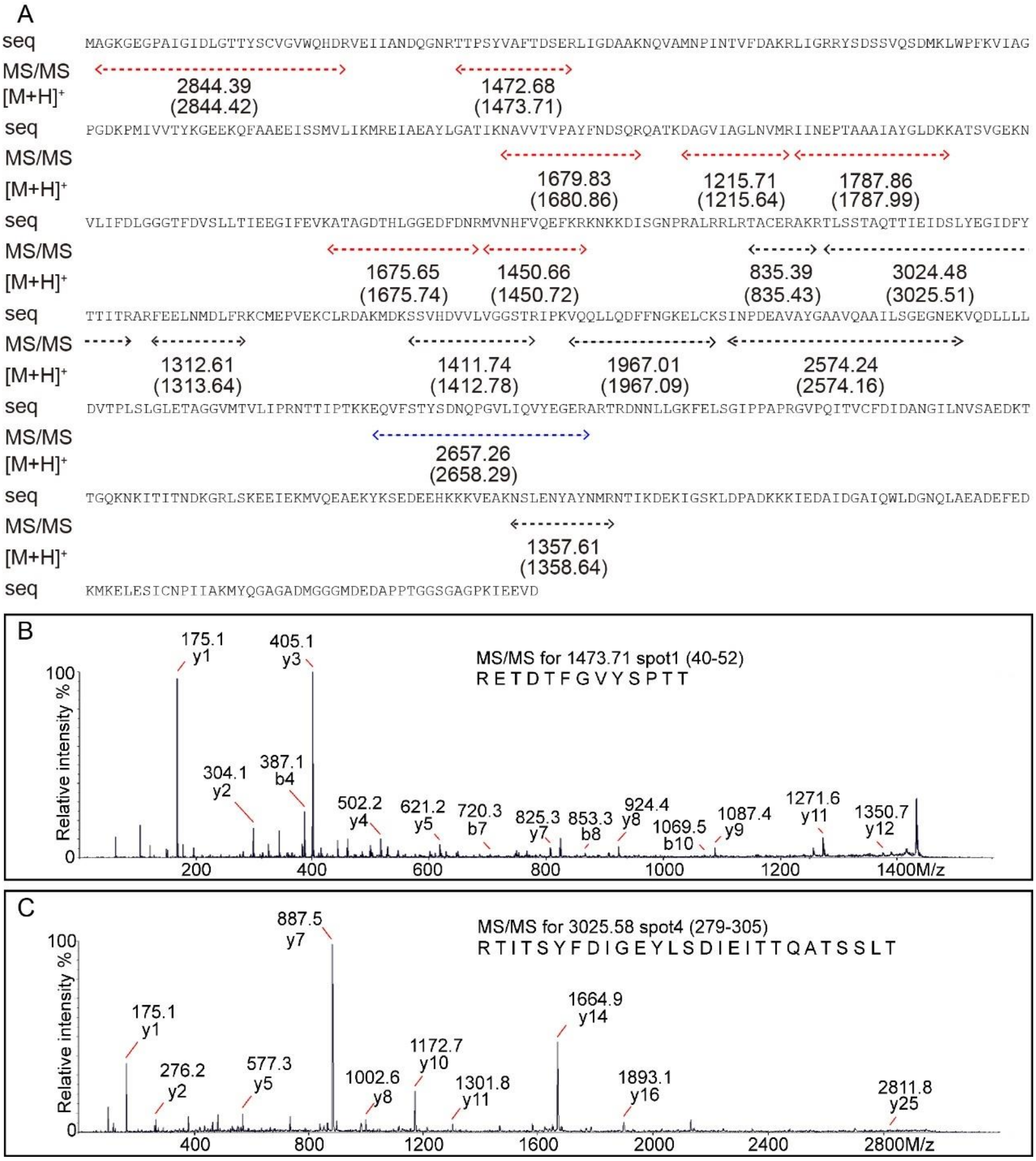

Supplementary Figure S3

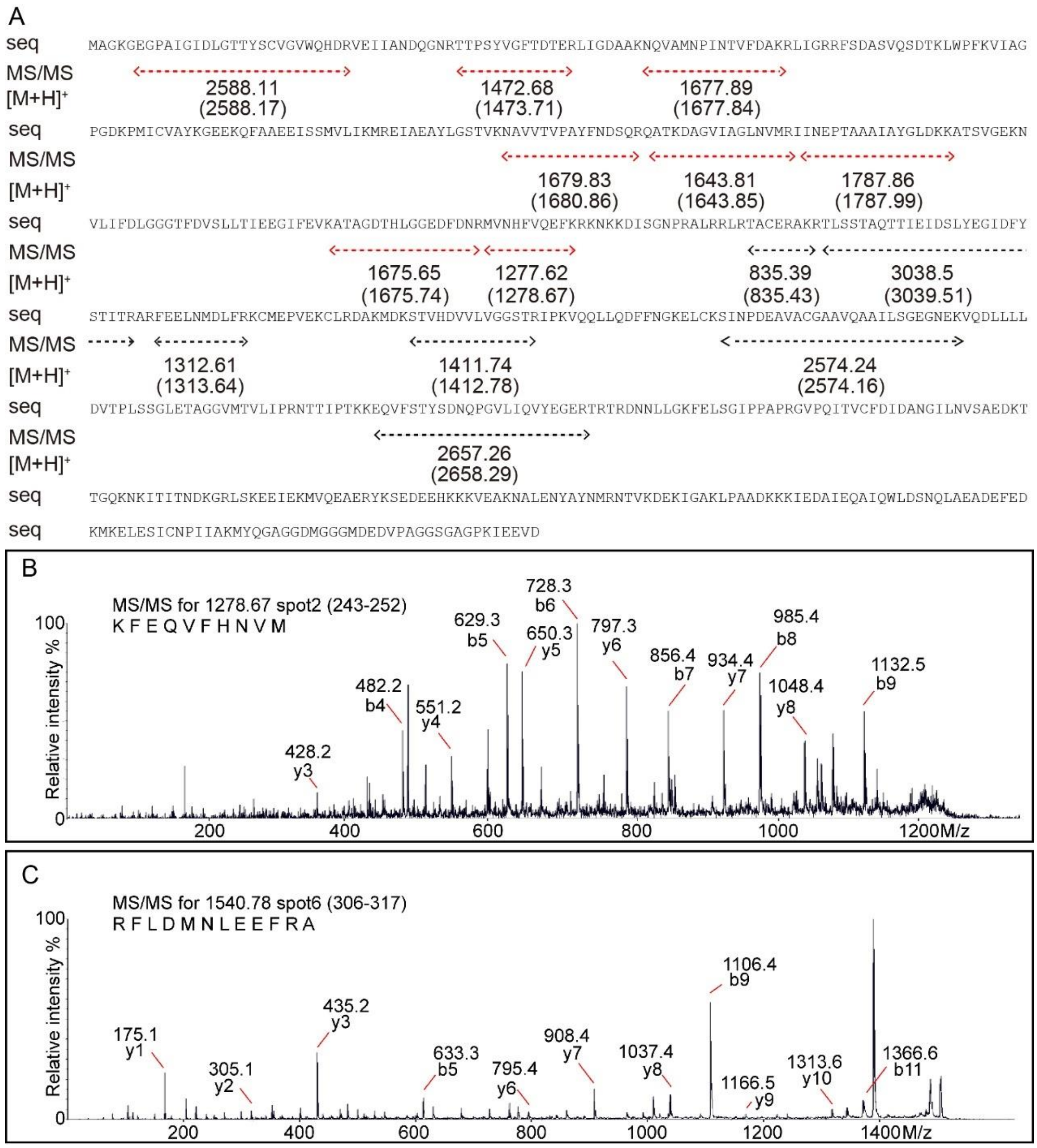

Supplementary Figure S4

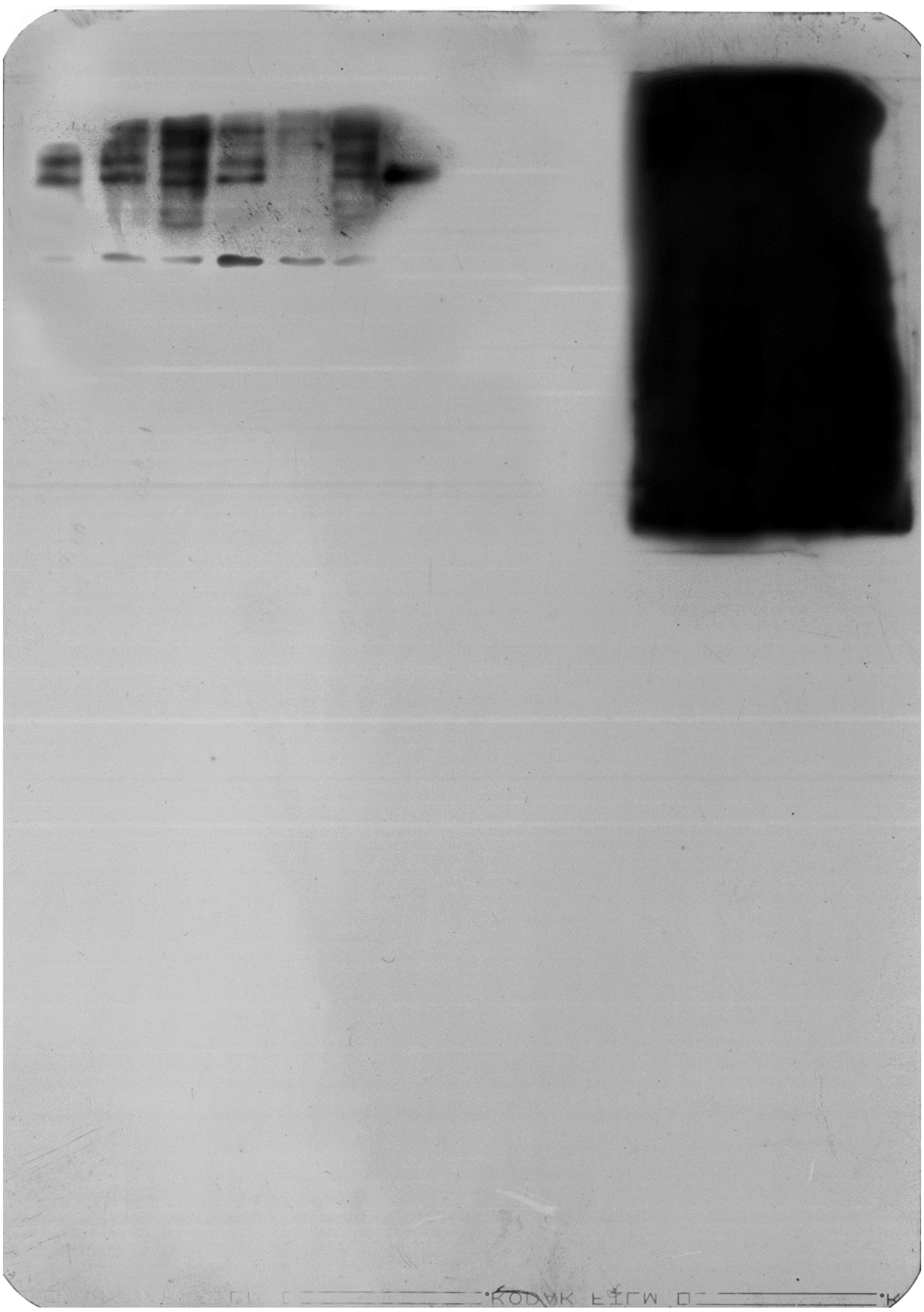

Supplementary Figure S5

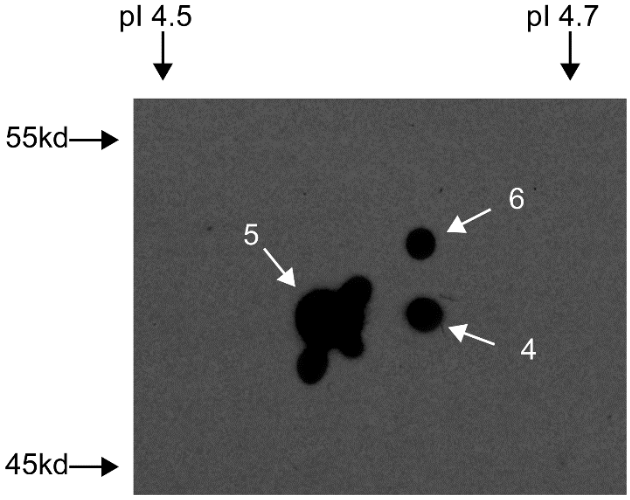

Supplementary Figure S6

A

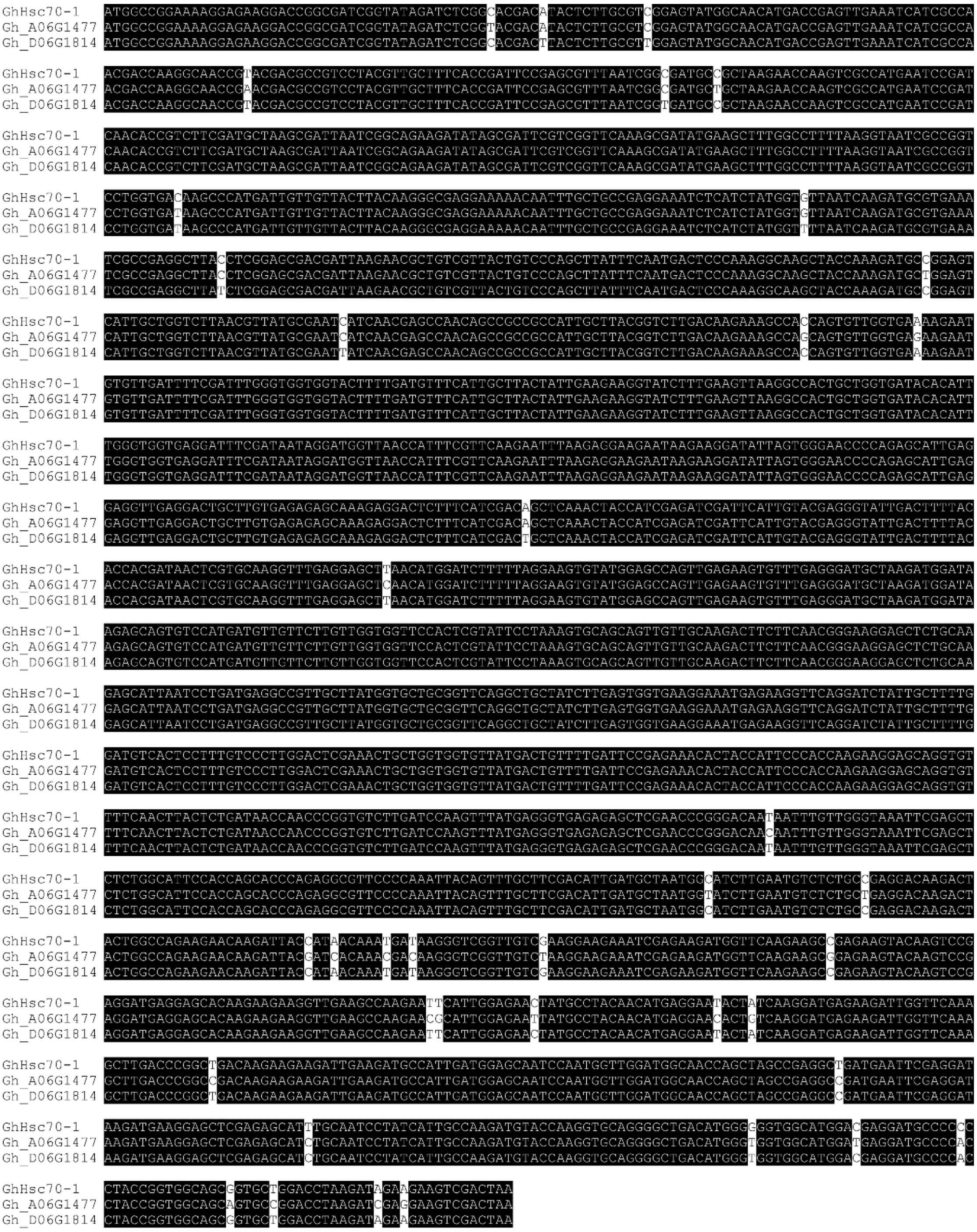

Supplementary Figure S6 (continued…)

B

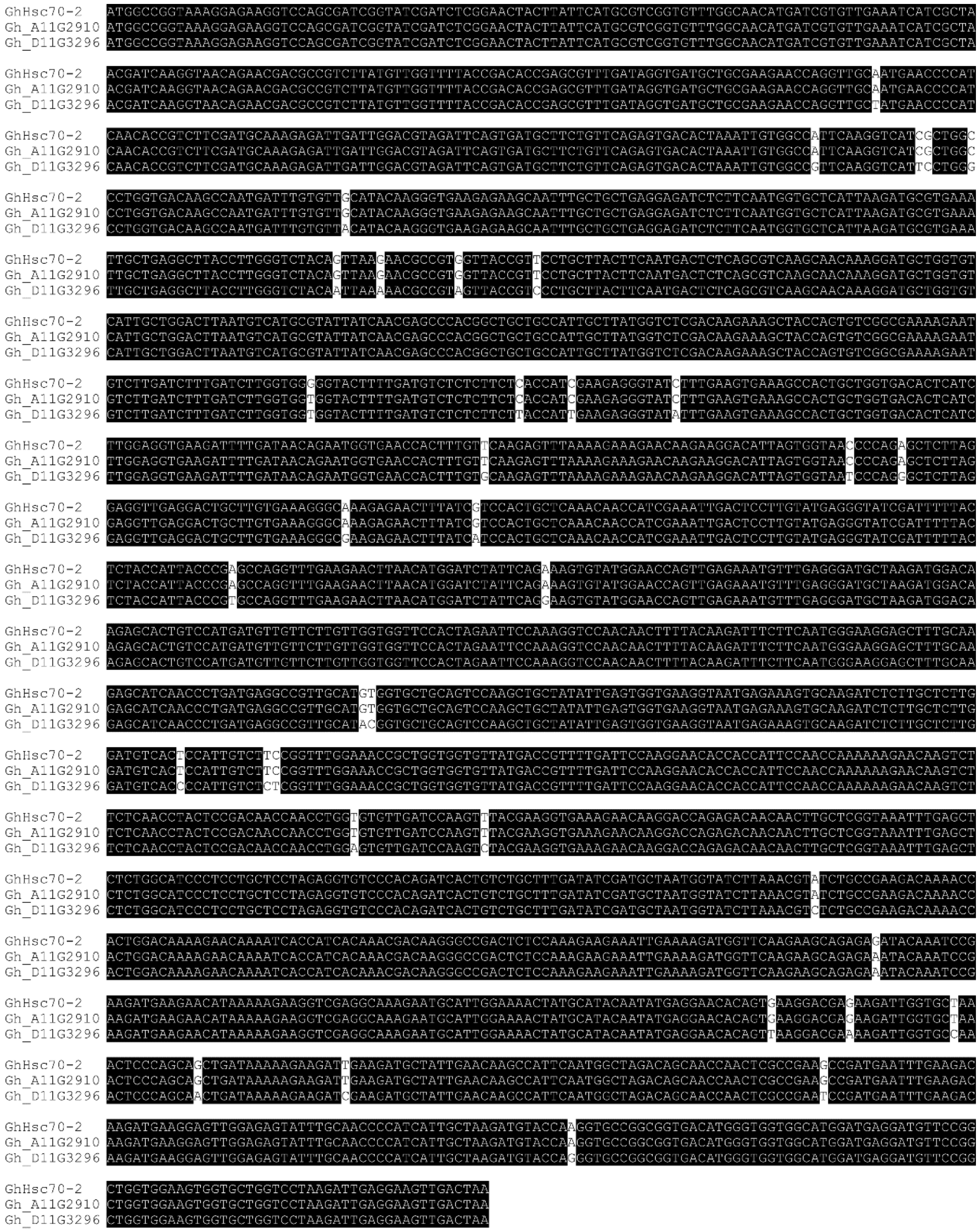

Supplementary Figure S6 (continued…)

C

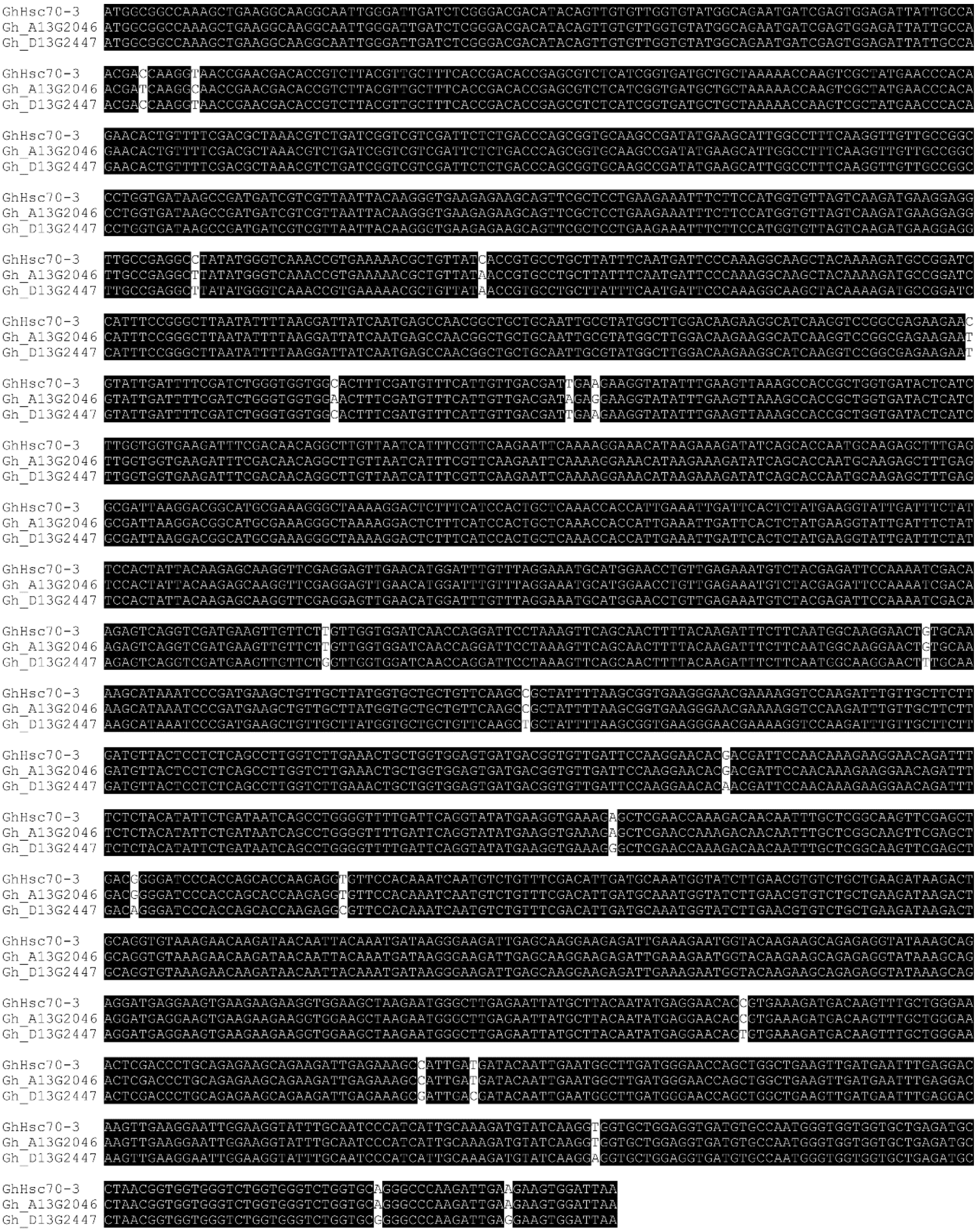

Supplement: Supplementary Information [file srep36961-s1.pdf]
